# Family disability, poverty and parenting stress: Analysis of a cross-sectional study in Kenya

**DOI:** 10.4102/ajod.v10i0.744

**Published:** 2021-06-10

**Authors:** Xanthe Hunt, Christina Laurenzi, Sarah Skeen, Leslie Swartz, Phillip Sundin, Robert E. Weiss, Mark Tomlinson

**Affiliations:** 1 Institute for Life Course Health Research, Department of Global Health, Faculty of Health Sciences, Stellenbosch University, Bellville, South Africa; 2 Department of Psychology, Faculty of Arts and Social Sciences, Stellenbosch University, Stellenbosch, South Africa; 3 Department of Biostatistics, UCLA Fielding School of Public Health, University of California, Los Angeles, United States of America; 4 School of Nursing and Midwifery, Queen’s University Belfast, Belfast, United Kingdom

**Keywords:** poverty, parent child relationship, parenting stress, disabled children, child development, child rearing

## Abstract

**Background:**

Households with a disabled member, be they a caregiver or a child, are poorer than households not affected by disability. Poverty, caregiving as a person with a disability and being the caregiver of a child with a disability can lead to increased parenting stress.

**Objectives:**

The objective of this study was to examine whether parenting stress experienced by caregivers in a household with a disabled member is greater when the disabled member is the caregiver, or the child, and how much of these respective relationships is explained by poverty.

**Method:**

We collected cross-sectional data using a demographic survey, the Washington Group Questions on adult disability, the 10 Questions on child disability and the Parenting Stress Index-Short Form, from 465 caregivers enrolled in a non-governmental child development programme in Kenya.

**Results:**

Households with a disabled member were poorer than households without a disabled member. Parenting stress of disabled caregivers was higher than parenting stress of non-disabled caregivers; however, this relationship disappeared when socio-economic status was controlled for. Caregivers of disabled children were more stressed than caregivers of non-disabled children, and this effect was not explained by differences in socio-economic status.

**Conclusion:**

Our findings highlight the importance of developing a comprehensive understanding of the stressors facing households with a disabled member, particularly if that member is a child, so that supportive interventions can adequately cater to the needs of caregivers, and their children, in the context of poverty.

## Introduction

There is a well-established relationship between disability and poverty (Emerson 2004; Groce et al. 2011; Mitra, Posarac & Vic 2013): disabled people are, on an average, poorer than non-disabled people. Although the precise relationship between disability and poverty is complex and varies from one context to another, there is evidence that disability may lead to impoverishment, and that poorer people are more likely to become disabled (Grech 2015; Mitra 2017). This relationship is true in low- and middle-income countries (LMICs), where disability is significantly associated with higher poverty (Mitra et al. 2013; Simeu & Mitra 2019), lower educational attainment, lower employment rates and higher medical expenditures (Mitra et al. 2013), as well as functional limitations (largely because of stigma and disabling environments) which make it difficult to attain the same standard of living as non-disabled people (Trani et al. 2015).

Disability may also have a profound impact on household relationships and developmental pathways. The associations between disability and parenting stress are well established (Anderson et al. 2007; Dyson 1993; Hassall, Rose & McDonald 2005; Rodrigue, Morgan & Geffken 2005), although evidence suggests that many intervening variables may influence this relationship. For example, it may be the factors surrounding disability, such as discrimination and access issues, rather than impairments per se, which lead to stress. Similarly, caregivers in poverty may experience more parenting stress than caregivers of higher socio-economic status (Brody et al. 1994; Raikes & Thompson 2005; Steele et al. 2016). What is less understood are the dynamics of parenting stress. For example, is increased stress experienced by caregivers of children with disabilities accounted for by the impact which disability may have on household income? Or, do the impacts of disability – either of the caregiver or the child – and associated factors like prejudice and barriers to access on parenting stress go beyond that of economic impact? Does this differ depending on who is disabled? Answers to these questions have not been established in concrete (although past studies have found varying associations; see Bannink, Idro & Van Hove 2016; Hassall et al. 2005; Meppelder et al. 2015).

The primary reasons that disability may lead to, or cement, poverty are threefold. Disabled people may have lower earning capacity, they may have accommodation needs because of their disability which create associated costs, and they may require assistance and caring by other family members which can lower the household’s overall earning capacity (Brown & Emery 2009; Meyer & Mok 2019; Montes & Halterman 2008). Poverty may lead to disability too; people living in poverty have less access to medical services than people of higher socio-economic status (Lustig & Strauser 2007) and may have poorer overall health (Anderson et al. 1997; Lochner et al. 2001) and increased risk of disability.

Disability is not an individual phenomenon, however, particularly as it relates to poverty (Palmer 2011). The reduced capacity to earn, additional expenses incurred and the requirements for care of these households mean that families with a disabled member are likely to have a lower standard of living than households of similar means, without a disabled member. This is particularly the case in low-income countries, where formal welfare systems and caring support services and infrastructure are limited. In these contexts, social capital in the form of informal care networks may buffer some of the deleterious effects of disability on the household’s socio-economic status, but this is unlikely to be to the extent necessary to negate the impacts of poverty. Families with a disabled member may not be able to yield as much social capital as other families, because of their relative lack of caring resources; their members are likely to be focused on the care of their own member, and less able to extend care to non-family members. As a result, these families may be at risk of social exclusion (Narayan-Parker & Patel 2000), and so unable to access these networks.

Emerson (2004) notes that the experience of poverty is likely to be associated with poor caregiver health and well-being and consequently poorer parenting practices. Caregivers of lower socio-economic status may experience more parenting stress than caregivers of higher socio-economic status (Katz et al. 2007; Silva et al. 2019), as certain dimensions of poverty such as material hardship and financial strain, and neighbourhood quality, contribute to high levels of stress amongst caregivers and may undermine their resilience to the ‘normal’ stresses of parenting (Cassells & Evans 2017). This is important because parenting stress is associated with poorer parenting practices, difficulties in the parent–child relationship and with poorer child adjustment (Deater-Deckard 2006). However, the strength of the relationship between poverty and parenting stress appears to be mediated by a variety of factors, including social support, trauma and parenting efficacy (Raikes & Thompson 2005; Steele et al. 2016).

Still, when low socio-economic status is compounded by the effects of having a disabled member in the household in a context which does not provide adequate support for people with disabilities, this stress might be greater. Both disabled caregivers and caregivers of children with disabilities are at risk of additional stress and may be in need of additional caregiving support (Craig et al. 2016; Hayes & Watson 2013).

However, it remains unclear whether the impacts of household disability on parenting stress differ depending on whether it is the caregiver or the child who is disabled, and how much of the relationship between parenting stress and disability is because of the relationship between disability and poverty. To begin to address these questions, in this study, we explore the relationship amongst disability, poverty and parenting stress in Western Kenya. Specifically, we examine whether parenting stress experienced by caregivers in a household with a disabled member is greater when the disabled member is the caregiver, or the child, and how much of these respective relationships is explained by household income. Understanding the relative role of income and socio-economic status in caregiving stress can help to inform policy and programming planning and design in LMIC, and ensure that the needs of caregivers and children in households affected by disability are reflected in interventions.

## Research methods and design

### Study design

We explored the relationship amongst disability, poverty and parenting stress using data from a large study of children enrolled in a parenting and early child development programme administered by Plan International, an international non-governmental organisation working in Western Kenya. The study was not originally designed for this analysis. However, given high rates of child and caregiver disability in the survey data set, we decided to explore associations between disability and socio-economic status and parenting stress, based on prior literature, suggesting the possibility of such relationships. We collected baseline cross-sectional (i.e. correlational) data from caregivers of children enrolled in Plan International’s Community-Led Action for Children programme.

### Setting

The study took place in Western Kenya, with data collected across predominantly rural sites in Kisumu, Homa Bay and Bondo Districts. Children and their caregivers were recruited through their attendance at early child development centres, which are typically part of government primary schools. Plan has provided support for 99 early child development centres across the area, expanding learning capacity and starting parenting groups affiliated with each centre that are open to all caregivers.

### Study population and sampling strategy

Plan International staff members in Kenya generated a list of all 99 early child development centres included in the programme. Each centre was rated as being low, average or high quality using a purpose-built rating tool. We randomly selected 20 early child development centres, equally distributed across these quality levels, as study sites. One of these centres was removed from the study after an incidence of community violence, resulting in a final sample of 19 centres.

Children were eligible for recruitment if they were attending one of these selected 19 early child development centres, and if they were 4 or 5 years of age at the time of baseline recruitment. All caregivers of eligible children were included in the study. The study team randomly selected and recruited an average of 30 caregivers per centre. Consent was obtained for the caregiver interviews. The final sample comprised 465 caregivers at baseline. In the present article, only data from the caregiver interview are used, as direct assessment of child disability was not performed.

### Measures

Caregivers were interviewed using a standardised questionnaire with two parts; one about the caregiver and household, and one about the child.

#### Demographics

We asked demographic questions about the caregiver, household and child. Questions about the household included household assets, electricity and water source, monthly income and number of household members. Questions about the caregiver included caregiver education, marital status, income sources, age, region where they live, relation with the child they care for, human immunodeficiency virus (HIV) status, monthly income and gender. For monthly income, we asked caregivers if they made above 2000 Kenyan Shillings (KsH) monthly, as 2000 KsH reflected the lowest stratum of income in the target communities (communities where almost all households fall below the USD defined poverty line, and as such, a comparative income bracket structure is needed to show variation between households). Questions about the child included age, HIV status, number of siblings and gender.

#### Caregiver disability

**Washington group questions:** The Washington Group Short Set of questions was used to identify disabled caregivers in the present study. The measure, which has been used in many contexts globally including Kenya (Altman 2016; Madans & Loeb 2013), was administered in survey format by trained data collectors on the project. Items include questions regarding the respondent’s functioning in seeing, hearing, ambulating, cognition, self-care and communication. It includes 10 items, which include questions such as ‘Do you have difficulty seeing, even if wearing glasses?’, which are answerable on a Likert scale with response options ranging from 1 (no, no difficulty) to 4 (cannot do at all). The cut-off employed here, as elsewhere (Loeb, Eide & Mont 2008), was any score of 3 or 4 on any one of the 10 items. To classify a caregiver as disabled, a binary outcome was created. A caregiver was marked as being disabled if he or she scored 3 or 4 on any of the possible disability outcomes.

#### Child disability

**Ten questions:** The ‘ten questions’ are used for the recognition of disabilities in community settings, and are a simple set of 10 items used to screen for disability in children (Durkin et al. 1991; Zaman et al. 1990). The caregiver report questionnaire contains 10 questions and probe questions that follow each of the questions. The questions are intended to be appropriate and useful for detecting disabilities in virtually all cultures and for all children aged 2–9 years. The checklist was translated into the local language. The questions are answerable yes/no, and include items such as ‘Compared with other children, does or did (child name) have any serious delays in sitting, standing or walking’?

A binary variable was created for child disability. If a child scored a ‘yes’ for any of the 10 disability-related questions asked, then that child was identified as disabled for our analysis.

#### Parenting stress

**Parenting stress index – Short form:** We employed the Parenting Stress Index – Short Form (PSI-SF) to gauge parenting practices and responsibilities stress (Abidin 1983). The 36 items include statements and prompts; the data collectors read the statement, and then prompt caregivers to respond with their level of agreement on a five-point Likert scale ranging from 1 (strongly disagree) to 5 (strongly agree). Higher scores on items after coding are indicative of higher levels of stress. This scale has been used in South Africa (Potterton, Stewart & Cooper 2007) as well as in West Africa (Guo et al. 2014) and Kenya (Oburu & Palmérus 2005). The outcome used is the total PSI-SF score, calculated as the sum of the items. We calculated a Cronbach’s alpha of 0.85.

### Data collection

All interviews and assessments were translated into Luo and Swahili. A trained team of experienced data collectors, who are local to the area, interviewed caregivers. The team held informational meetings at each site for caregivers and teachers ahead of data collection at each school. Prior to individual interviews with caregivers, which were scheduled via cell phone ahead of time, data collectors obtained informed consent from caregivers. These forms were securely stored with the on-site supervisor (X.H. or C.L.), before being transferred to (Stellenbosch University) at a later date.

All data collectors were fluent in Luo and Swahili, and conducted interviews in the caregiver’s preferred language. All survey responses were collected by the data collectors using a tablet-based data collection application. Data were stored on password-protected computers once downloaded from the application’s cloud.

## Data analysis

Data analysis was structured to address our different areas of interest:

- Firstly, caregiver disability was examined for its association with several key demographic variables, including caregiver, child and household characteristics to establish the relationship of household disability with poverty. As these relationships are using counts of caregivers in each category, we use chi-squared tests to determine any potential associations.
- We then ran univariate linear regressions for each predictor on parenting stress, measured by the total score of the caregiver on the PSI-SF measure (PSISum).
- We then chose a subset of the significant demographic predictors to include in a multiple linear regression based on what predictors were significant in the univariate regression. The demographics selected were chosen to best represent socio-economic status based on household item ownership and household monthly income. Controlling for these demographic variables, we examined the relationship between caregiver disability and parenting stress.
- We then performed a similar analysis using child disability as a predictor of parenting stress (again controlling for the demographic variables).
- In the final multiple linear regression, we included both caregiver and child disability.

For analyses that included monthly income as a predictor, we dropped observations that did not report income. All analyses were performed in R, and a 0.05 level of significance was used across analyses (R Core Team 2017).

### Ethical considerations

The authors assert that all procedures contributing to this work comply with the ethical standards of the South African and Kenyan national and institutional committees on human experimentation and with the Declaration of Helsinki 1975, as revised in 2008. Ethical approval for the study was obtained from the ethics boards at Stellenbosch University (N15/10/099) and the Ethics and Scientific Review Committee at AMREF in Kenya (P220/2016).

## Results

Table 1 presents the demographic characteristics of caretakers, households and children. Our sample included 465 caregivers who reported information about themselves and their children (*n* = 497 children). Nearly 90% of the caregivers in the sample were women and 27% of the caregivers were disabled.

**TABLE 1a:** Demographic information.

Caregiver characteristics (*n* = 465)	Disabled caregiver (*n* = 126)		Not disabled caregiver (*n* = 339)	
	*n*	(% of disabled)	*n*	(% of not disabled)
**Caregiver gender (*χ*^2^ = 5.72, *p* = 0.02)**				
Male	7	6	48	14
Female	119	94	291	86
**Caregiver education (*χ*^2^ = 206, *p* < 0.001)**				
No school	47	37	6	2
Grades 1–6	58	46	48	14
Grade 7–8	11	9	197	58
Grades 9 and above	10	8	88	26
**Caregiver marital status (*χ*^2^ = 3.67, *p* = 0.055)**				
Not married	15	12	22	6
Married	111	88	317	94
**Caregiver HIV status (*χ*^2^ = 0.001, *p* = 0.9703)**				
Positive	29	23	76	22
Negative	93	74	246	73
Not available	4	3	17	5

**TABLE 1b:** Demographic information.

Household characteristics (*n* = 465 caregivers)	*n*	(% of Disabled)	*n*	(% of not disabled)
**Household members count (*χ*^2^ = 0.072, *p* = 0.788)**				
0–5	67	53	185	55
6+	59	47	154	45
**Household monthly income (*χ*^2^ = 3.93, *p* = 0.045)**				
0–2000 Kenyan shillings	79	63	193	57
2001+ Kenyan shillings	30	24	119	35
Not available	17	13	27	8
**Household assets prevalence**				
**Cell phone ownership (*χ*^2^ = 5.54, *p* = 0.018)**				
No cell phone	10	8	10	3
Has cell phone	116	92	329	97
**Radio ownership (*χ*^2^ = 21.90, *p* < 0.0001)**				
No radio	61	48	87	26
Radio	65	52	252	74
**Electricity (*χ*^2^ = 5.61, *p* = 0.02)**				
No electricity	49	14	8	6
Electricity	290	86	118	94
**Bicycle ownership (*χ*^2^ = 4.197, *p* = 0.04)**				
No bicycle	93	74	216	64
Bicycle	33	26	123	36
**Stove ownership (*χ*^2^ = 9.17, *p* = 0.002)**				
No stove	105	83	235	69
Stove	21	17	104	31
**Internet access on phone (*χ*^2^ = 7.11, *p* = 0.007)**				
No Internet (phone)	108	86	251	74
Internet (phone)	18	14	88	26
**TV ownership (*χ*^2^ = 2.58, *p* = 0.1076)**				
No TV	117	93	297	88
TV	9	7	42	12

**TABLE 1c:** Demographic information.

Child characteristics (*n* = 497 children)	*n* = 303 children without a disability		*n* = 194 children with a disability	
	*n*	(% of not disabled)	*n*	(% of disabled)
**Child’s age (months) (*χ*^2^ = 3.00, *p* = 0.8)**				
48–59	154	50.8	114	58.8
60–72	149	49.2	80	41.2
**Disciplinary responsibility (*χ*^2^ = 0.04, *p* = 0.83)**				
Female caregiver	254	83.8	164	84.5
Male caregiver	49	16.2	30	15.4
**Child HIV status (*χ*^2^ = 5.00, *p* = 0.8)**				
Negative	247	81.5	149	76.8
Positive	2	0.6	6	3.1
Not available	54	17.8	39	20.1

HIV, human immunodeficiency virus.

Our sample had 258 male children (52%) and 239 female children (48%). Thirty-nine per cent of the children were disabled. There were about equal numbers of children who were 4 years of age, defined as being aged between 48 and 59 months (*n* = 268, 54%) and 5 years, defined as being aged between 60 and 72 months of age (*n* = 229, 46%). Of all caregivers, 24% were HIV-positive and 1.6% of all children were HIV-positive, using a self-report measure of sero-status.

Amongst caregivers, 29% of female caregivers were disabled as compared to 13% of men (*p* = 0.02). There also appeared to be an association between caregiver education and disability, as disabled caregivers tended to be less educated than non-disabled caregivers (*p* < 0.0001). Although not shown, a chi-squared test was also run to examine any association between child gender and child disability, but no relation found (χ^2^ = 0.00; *p* = 1.00). Of the caregivers who reported monthly income (*n* = 421), 65% of caregivers made below the 2000 KsH monthly threshold. However, there existed a disparity depending on caregiver disability. Of the disabled caregivers who reported income (*n* = 109), 72% were below the threshold, compared to 62% of non-disabled caregivers who reported income (*n* = 312; *p* = 0.05).

In Table 2, caregiver disability significantly predicted a higher total PSI score in the univariate linear regression, indicating greater parenting stress (*p* < 0.001, see Table 2). Caregiver education, a monthly income above the 2000 KSh threshold, cell phone ownership, bicycle ownership, stove ownership and television ownership were all significantly negatively associated with parenting stress, whilst a higher number of members in the household was positively associated with parenting stress. Education, household member count and monthly income were all included in the final multiple regression analysis; only cell phone ownership was included amongst the household items because it was the only asset which showed variation (with bicycle ownership and radio ownership, for instance, being equivalent between groups and very common), and was sufficient to capture the item ownership aspects of socioeconomic status (SES). After controlling for cell phone ownership, income, education and number of members in the household (SES), caregiver disability was no longer significant (*p* = 0.15, see Table 3).

**TABLE 2:** Demographic and caregiver disability linear regression, Parenting Stress Index-Short Form measure as outcome.

Univariate analysis (*n* = 465, all caregivers)	Coefficient	Standard error	*t*	*p*
Female caregiver	0.46	1.27	0.37	0.7100
Married	−2.34	1.51	−1.55	0.1200
Education	−0.70	0.14	−4.91	< 0.0001
Number of household members	0.54	0.22	2.54	0.0100
Above 2000 KSh monthly income	−4.48	0.87	−5.14	< 0.0001
Cell phone ownership	−6.91	2.00	−3.46	0.0005
Radio ownership	−1.58	0.88	−1.81	0.0700
Bicycle ownership	−2.18	0.86	−2.53	0.0100
Stove ownership	−2.41	0.91	−2.68	0.0070
Internet access on phone	0.94	0.97	0.97	0.3300
TV ownership	−3.77	1.29	−2.90	0.0030
Electricity	2.18	1.246	1.75	0.0800
Caregiver disability	3.25	0.91	3.59	< 0.0001
Child disability	3.96	0.81	4.86	< 0.0001

KSh, Kenyan shillings.

**TABLE 3:** Demographic and caregiver disability multiple linear regression, Parenting Stress Index-Short Form measure as outcome.

Variable	Coefficient	Standard error	*t*	*p*
**Multiple analysis (*n* = 421, those who reported income)**				
Caregiver disability	1.42	0.98	1.46	0.1500
Cell phone ownership	−5.11	2.16	−2.36	0.0200
Above 2000 KSh monthly income	−3.85	0.88	−4.40	< 0.0001
**Education overall (reference no school) *F* = 2.43, *p* = 0.06**				
1st to 6th grade caregiver education	1.47	2.70	0.54	0.5800
7th and 8th grade caregiver education	−0.97	2.63	−0.37	0.7100
9th grade and above caregiver education	−1.94	2.74	−0.71	0.4800
Number of household members	0.50	0.21	2.33	0.0200

KSh, Kenyan shillings.

In the univariate linear regression, child disability was significantly predictive of greater parenting stress (*p* < 0.002, see Table 2). After controlling for the same demographics used in the multiple regression model for parental disability, child disability remained a significant predictor (*p* < 0.0001, see Table 4). Caregiver disability was not significant. Other covariates that remained significant were cell phone ownership, monthly income and the number of household members. Having a cell phone and higher income were associated with lower parental stress, and more members in the household were associated with higher stress.

**TABLE 4:** Demographic, caregiver and child disability multiple linear regression, Parenting Stress Index-Short Form measure as outcome.

Variable	Coefficient	Standard error	*t*	*p*
**Multiple analysis (*n* = 421, those who reported income)**				
Child disability	3.44	0.83	4.15	< 0.0001
Caregiver disability	1.17	0.96	1.22	0.2200
Cell phone ownership	−5.36	2.12	−2.53	0.0100
Above 2000 KSh monthly income	−3.63	0.86	−4.22	< 0.0001
**Education *F* = 2.17, *p* = 0.09**				
1st to 6th grade caregiver education	2.09	2.66	0.79	0.4300
7th and 8th grade caregiver education	−0.05	2.59	−0.02	0.9800
9th grade and above caregiver education	−1.17	2.70	−0.43	0.6600
Number of household members	0.45	0.21	2.13	0.0300

KSh, Kenyan shillings.

## Discussion

In this study, we found that households with a disabled member were poorer than households without a disabled member. Parenting stress amongst disabled caregivers was higher than parenting stress amongst non-disabled caregivers; however, this relationship disappeared when socio-economic status was controlled for. Finally, caregivers of disabled children were more stressed than caregivers of non-disabled children, and this effect was not explained by variance in socio-economic status, that is, the association between child disability and parenting stress was not attributable to the association between child disability and socio-economic status.

The fact that disability was associated with poverty at the household level is to be expected, given past research (Braithwaite & Mont 2009; Emerson 2004; Lustig & Strauser 2007). The finding regarding caregiver disability and parenting stress is interesting, however. The impact of caregiver disability on parenting stress appears to be largely because of the association between disability and income, suggesting that disabled caregivers are experiencing greater parenting stress because of their relative poverty, rather than the impact of their impairment on their capacity to take care of children.

On the other hand, the fact that the association between child disability and parenting stress was not explained entirely by the association between disability and income suggests that the stressors faced by caregivers of children with disabilities go beyond the impact of disability on household socio-economic status. Factors such as inaccessible schooling, inaccessible healthcare services and a dearth of other resources with which to attend to the child’s needs – needs that are not unrealistic, but simply at odds with that which the environment in rural Kenya can provide for – place a burden of stress on these caregivers, which exceeds financial strain. A recent study from Uganda found that caregivers of children with disabilities experienced extremely high levels of parenting stress, which, the authors suggested, was because of the functional limitations of the child (and thus burden of care), as well as stigma (Bannink et al. 2016). In this study, we did not ask disability-specific stigma or social isolation questions, and so conclusions in this regard cannot be drawn. However, given the extra burden of work placed on caregivers of children with disabilities in contexts of low social support and little infrastructural safety net, it is possible that these caregivers are socially isolated. Past studies by Gupta (2007) and Ones et al. (2005) have found elevated rates of social isolation amongst caregivers of children with developmental conditions. Coupled with disability stigma, which has been found in past studies in Kenya, these caregivers may face layers of marginalisation which compounds the negative effects of poverty (Gona et al. 2011; Monk & Wee 2008).

It is important to note that this study has several limitations engendered by the fact that this analysis was not the primary interest of the original study. Firstly, the disability measures used were not optimal (observational tools would have been preferable), but the main study from which these data are drawn was not centrally concerned with disability, and so self-report tools were selected, to minimise total interview and assessment time for parent–child dyads. Secondly, a potential limitation of the study is the fact that the children included in this sample were already attending an Early Child Development (ECD) centre, which puts them at an advantage over most children with disabilities who may not be able to attend school because of various reasons including poverty. Therefore, in this study, the income level of caregivers may have been slightly higher than the rest of caregivers with children with disabilities at home, and the level of disability amongst children may have been overestimated (given the estimated prevalence of 39% children with some disability) compared to the true population of children with disabilities.

Taken together, these findings clearly point to the need for additional support for families with a disabled member, particularly disabled children. Our findings show that these households are poorer than households without a disabled member. Cash transfers and social support have been implemented with much success to support families affected by disability in Brazil (Lindert et al. 2007; Medeiros, Diniz & Squinca 2015) and to some extent in South Africa (Mitra 2010). The Brazilian model, where support is provided to the whole family, may be more suitable to the Kenyan context, given the present findings of family-level impact of disability on household poverty.

Furthermore, early child development non-governmental organisations need to have supports in place to ensure that disability-related programming, often a feature of their programming, is implemented. Non-governmental organisations and community-based organisations working with children should have greater engagement with caregivers in households affected by disability, and be cognisant of parenting stress as a factor which affects families’ capacity to cope with any implications of the individual’s disability. Importantly, programming should be more broadly focused on supporting caregivers to cope positively in the context of child disability (including stress management for caregivers with children with disabilities) rather than focusing on the child as the ‘cause’ of parenting stress.

Emerson (2004) wrote that:

[*I*]t will be important for organisations providing services to children with intellectual disabilities and their families to: (1) explicitly recognise the importance of child poverty in defining the family resources that shape the context in which services and supports need to be delivered; (2) forge links between services for children with intellectual disabilities and their families and local initiatives aimed at either eradicating or reducing the impact of poverty. (p. 332)

Despite the distance in time from Emerson’s (2004) observation, and expanding it to include physical and not only intellectual disability, this point still holds. Our findings suggest that caregivers of children with disabilities face stressors that exceed the impacts of poverty on their functioning and well-being. This highlights the importance of comprehensive needs assessments and situational analyses being conducted by prospective programmers in these settings, as without a nuanced understanding of these stressors, supportive interventions may not adequately cater to the needs of caregivers, or their children.
